# BRCA1 and STMN1 as prognostic markers in NSCLCs who received cisplatin-based adjuvant chemotherapy

**DOI:** 10.18632/oncotarget.20715

**Published:** 2017-09-08

**Authors:** Mingxing Wang, Wanjun Li, Xuemei Xing, Dan Zhang, Jie Lei, Guoyin Li

**Affiliations:** ^1^ Department of Thoracic Surgery, Tangdu Hospital, The Fourth Military Medical University, Xi’an, Shaanxi, China; ^2^ Department of Pathology, Hanzhong 3201 Hospital Affiliated to Xi’anJiaotong University, Xi’an, Shaanxi, China; ^3^ Department of Clinical Laboratory, Hanzhong 3201 Hospital Affiliated to Xi’anJiaotong University, Xi’an, Shaanxi, China; ^4^ Department of Oncology, Hanzhong 3201 Hospital Affiliated to Xi’anJiaotong University, Xi’an, Shaanxi, China; ^5^ Department of Biochemistry and Molecular Biology, The Fourth Military Medical University, Xi’an, Shaanxi, China

**Keywords:** NSCLC, BRCA1 and STMN, chemotherapy, prognosis

## Abstract

**Objective:**

In this study, we aimed to investigate the predictive effect of *BRCA1*, *STMN1*, *MAPT* and *TUBB3* on the prognosis of patients with non-small cell lung cancer (NSCLC).

**Methods:**

Seventy NSCLC patients who received platinum-based chemotherapy from June 2009 to July 2011 were enrolled. The protein and mRNA levels of *BRCA1*, *STMN1*, *MAPT* and *TUBB3* were determined. Survival time of the patients with NSCLC was also calculated.

**Results:**

High expression of *BRCA1* or low expression of *STMN1* was associated with a better prognosis in NSCLC patients (*p*<0.01). In contrast, the expression of *MAPT* and *TUBB3* were not closely related with the prognosis of NSCLC patients(*p*>0.05). Furthermore, patients with high expression of *BRCA1* and low expression of *STMN1* have lived longer (*p*<0.01).

**Conclusion:**

*BRCA1* and *STMN1* were independently predictors for prognosis of NSCLCs which received cisplatin-based adjuvant chemotherapy.

## INTRODUCTION

Lung cancer is one of the most frequent malignant neoplasm and it was the main cause of cancer-related death all over the world [1]. NSCLC accounts for approximately 85% of all lung cancer. Moreover, most of the patients diagnosed with NSCLC have advanced local invasion and/or distant metastases at the time of diagnosis. Despite of recent improvement in surgery, chemotherapy, radiotherapy techniques, the long-term time of NSCLCs is still not satisfied. According to previous studies, the 5-year survival rate of NSCLCs stayed at approximately 16% and was accompanied by high recurrence rate, it has no significant improvement during the last two decades [2–4]. The high morbidity and mortality of NSCLCs made a great threat to the health and life of populations.

Platinum and taxane were extensively used [5–8], and platinum-based double drug combination as a standard treatment for patients with a good performance status scale had been recommended by 2011 guidelines of the National Comprehensive Cancer Network (NCCN). Platinum agents bind to DNA and form complexes with DNA, thus inhibiting the cell cycle and promoting apoptosis [9]. Taxanes bind to tubulin and suppress spindle microtubule dynamics, which leads to cell cycle arrest in G2/M phase followed by apoptosis [10]. However, NSCLCs with similar clinical pathology characteristics exhibit considerable heterogeneity to chemotherapy in sensitivity and toxicity. Primary and secondary drug resistance is the major cause of the problem [11, 12].Therefore, it is crucial to investigate efficient markers that can predict sensitivity or toxicity tolerance to chemotherapy.

Previous studies have reported that various biomarkers such as excision breast cancer 1 (BRCA1), β-tubulin-III (TUBB3), microtubule-associatedprote in tau (MAPT) and Stathmin (STMN1), which were closely associated with the clinical effect of NSCLCs undergoing chemotherapy [13–16].

Platinum-induced apoptosis can be identified and repaired via the nucleotide excision repair (NER) and base excision repair (BER) pathways [17]. ERCC1 and BRCA1 are critical proteins in the NER pathway and act as rate-limiting enzymes [18]. Paclitaxel binds to TUBB3, thereby inhibiting the cell cycle and leading to cell cycle arrest in G2/M phase [19]. MAPT primarily functions as tubulin assembly and microtubule stabilization and it can bind to the paclitaxel-binding site on the inner surface of the microtubule [20]. STMN1 can modulate microtubule dynamics through preventing polymerization of tubulin and boosting destabilization and disassembly of microtubule during the interphase and late mitosis along cell cycle progression [21].

Analysis of biomarkers could predict the sensitivity/toxicity to chemotherapy, which is very important for precision medicine and is widely accepted. It could significantly improve the survival and/or quality of life of NSCLCs. Overall, these capable biomarkers had been demonstrated as prognostic and predictive markers in certain studies, but not in others [15–19]. Accordingly, the present prospective, randomized, non-interventional study was performed to verify the predictive value of BRCA1, TUBB3, MAPT and STMN1 in patients with NSCLC that received adjuvant platinum-based chemotherapy.

Our study included 70 NSCLC patients who received platinum and paclitaxel based chemotherapy. The expression level of STMN1, BRCA1, MAPT and TUBB3 was detected by immunohistochemistry. We also studied the effect of the target genes above on the survival time of the NSCLC patients.

## RESULTS

### The expression of BRCA1 and MAPT in NSCLC decreased, but the expression of STMN1 and TUBB3 increased

**Figure 1:**
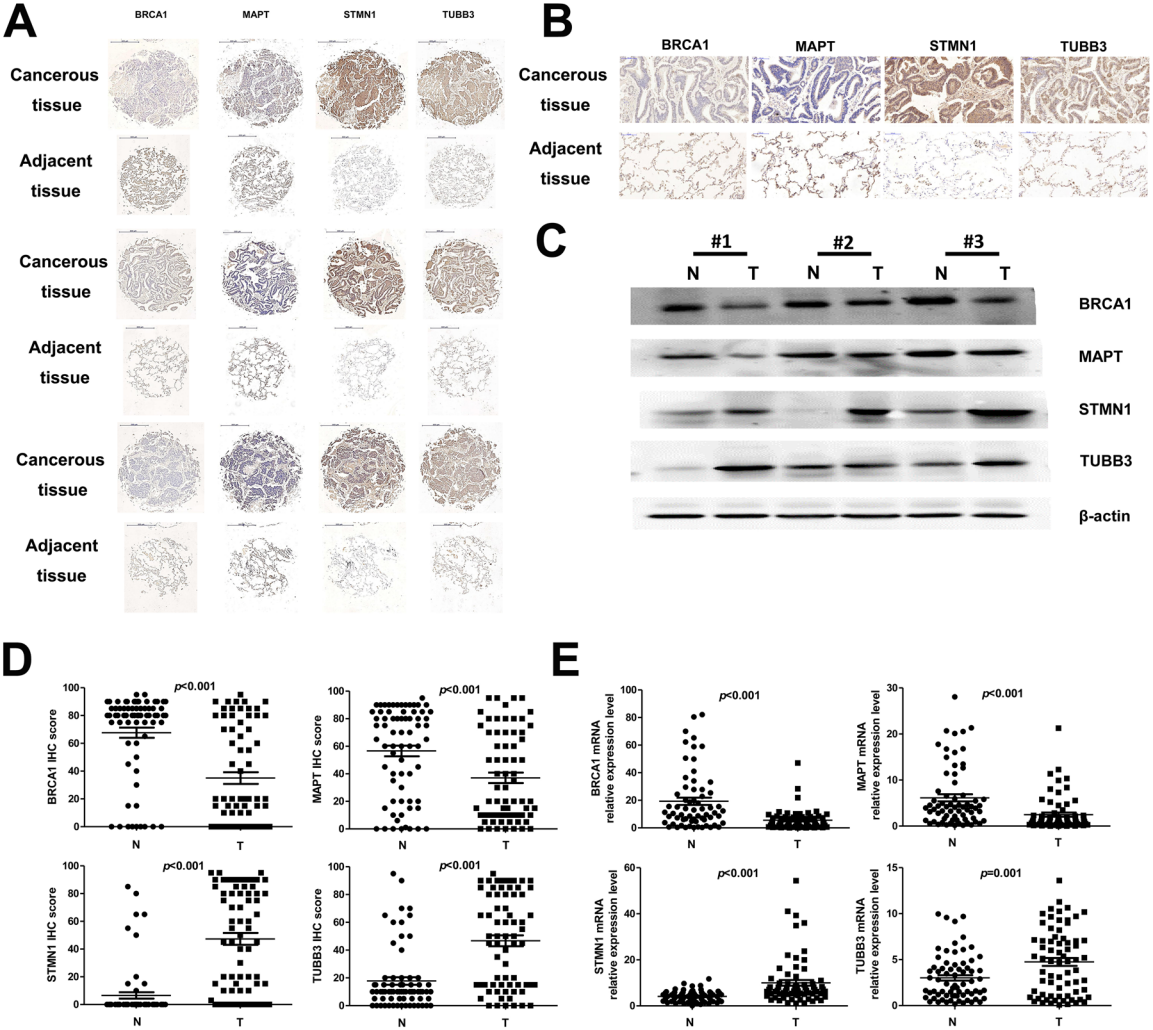
BRCA1, STMN1, MAPT and TUBB3 protein and mRNA expression levels in cancer tissues and adjacent nontumor tissues **(A-C)** Detection of protein levels in NSCLC tissues and adjacent non-tumor tissues. (A and B) Immunohistochemical assays, (C) western blot analysis. **(D)** Scatter plot of the tissue chip analysis of clinical samples. **(E)** Scatter plot of the mRNA expression level analysis of clinical samples. N: adjacent non-tumor tissue, T: cancer tissue.

70 pairs of NSCLC cancer tissues and adjacent tissues were made into tissue microarray. Protein and RNA samples were extracted with fresh clinical samples. IHC was used to detect the protein expression levels of BRAC1, STMN1, MAPT and TUBB3 in cancer tissues and adjacent non-tumor tissues (Figure 1A, 1B). The expression levels of target proteins from part of the clinical samples were detected by western blot (Figure 1C). Tissue microarray statistical results showed that the expressions of BRCA1 and MAPTin cancer tissues were significantly lower (*p*<0.01) than that in adjacent tissues, whereas the expressions of STMN1 and TUBB3 in cancer tissues were significantly higher (*p*<0.01) than that in adjacent tissues (Figure 1D). The mRNA expression levels of target genes were in accord with the expression of the protein (Figure 1E).

### There were no significant differences in the expression of BRAC1, STMN1, MAPT and TUBB3 in different age groups

**Figure 2:**
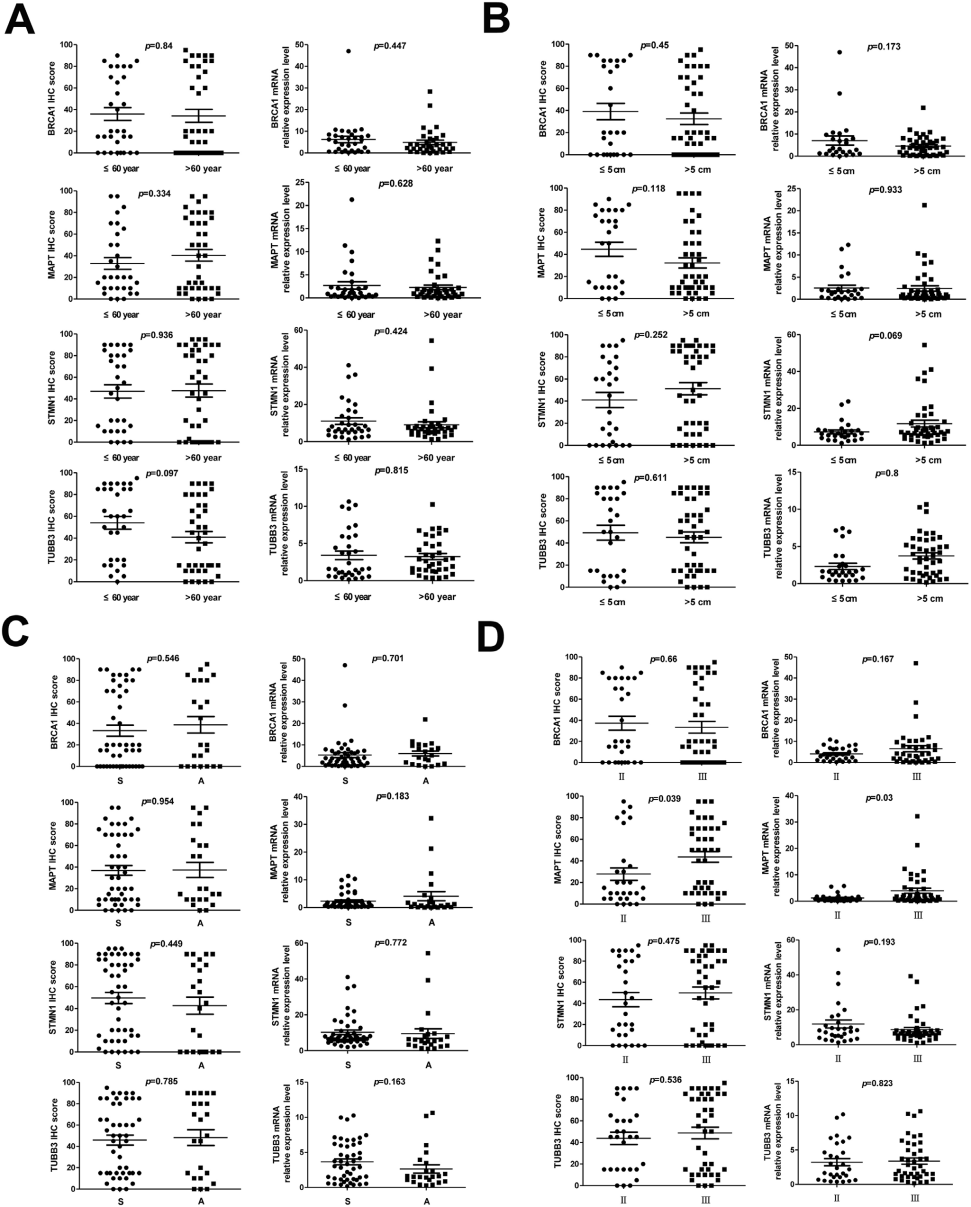
Analysis of the correlation between clinical features and expression levels of BRCA1, MAPT, STMN1 and TUBB3 in NSCLC **(A-D)** The scatter plots of the influence of clinical features on the expression of target genes, onset age (A), tumor size (B), pathological type (C), pathological stage (D). Protein (left), mRNA (right), S: squamous cell carcinoma, A: Adenocarcinoma.

According to the average onset age (60) of the patients, this study divided them into two groups. Protein and mRNA expression levels of BRCA1 (*p*=0.84 and *p*=0.447), STMN1 (*p*=0.936 and *p*=0.424), MAPT (*p*=0.334 and *p*=0.628) and TUBB3 (*p*=0.097 and *p*=0.815) in the both groups were detected by the methods mentioned above, and no significant difference was found (Figure 2A).

### The tumor size had no significant effect on the expression of BRAC1, STMN1, MAPT and TUBB3

According to the maximum diameter of the tumor, this study divided them into two groups: patients with bigger tumors (maximum diameter >5) and patients with smaller tumors (maximum diameter ≤5). Our study showed that tumor size had no significant effect on the expression (protein and mRNA) of BRAC1(*p*=0.45 and*p*=0.173), STMN1(*p*=0.252 and*p*=0.069), MAPT(*p*=0.118 and*p*=0.933) and TUBB3 (*p*=0.611 and*p*=0.8) between the two groups (Figure 2B).

### The expression level of BRAC1, STMN1, MAPT and TUBB3 in lung adenocarcinoma and squamous cell carcinoma showed no significant difference

This study included 47squamous cell carcinoma and 23 adenocarcinoma patients. Our results showed no significant difference in the protein and mRNA expression of BRCA1 (*p*=0.546 and *p*=0.701), STMN1 (*p*=0.449 and *p*=0.772), MAPT (*p*=0.954 and *p*=0.183) and TUBB3 (*p*=0.785 and *p*=0.163) between squamous cell carcinoma and adenocarcinoma (Figure 2C).

### The expression of MAPT was higher in stage III than that in stage II

All the patients included in this study were with stage II or β. Theprotein and mRNAexpression levels of MAPT were significantly lower in the patients with stage II than that in patients with stage III(*p*=0.039 and *p*=0.03, Figure 2D).However, our study did not find significant difference in the expression (protein and mRNA) of BRCA1(*p*=0.66 and*p*=0.167), STMN1(*p*=0.475 and*p*=0.193) and TUBB3(*p*=0.536 and *p*=0.823) in patients with stage IIand patients with stage III.

### The effect of target genes expression levels and clinical features on the 5 year survival rate of NSCLC patients

**Figure 3:**
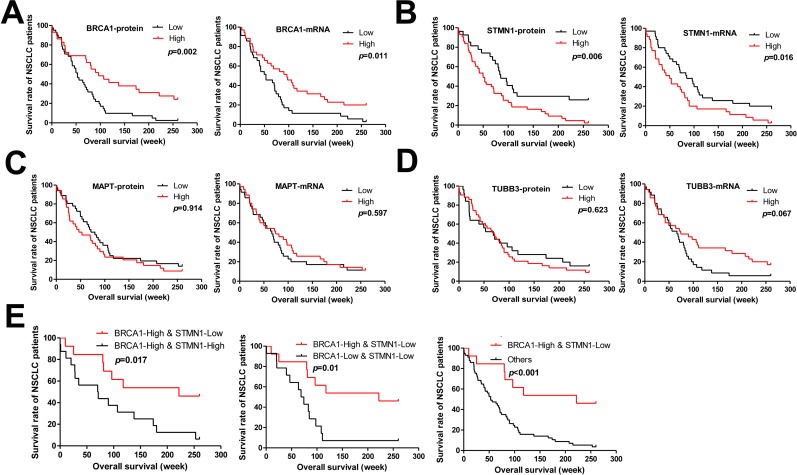
Kaplan–Meier curves with univariate analyses (log rank) of 70 NSCLC patients after surgical resection revealed 5-year survival **(A-D)** Patients were grouped according to the expression of BRCA1, STMN1, MAPT and TUBB3 in the carcinomas, and subjected to follow-up investigations. The percent of surviving patients was plotted, BRCA1 (A), STMN1 (B), MAPT (C), TUBB3 (D). **(E)** The prognostic accuracy of BRCA1 combined with STMN1 is higher than that of either alone.

**Figure 4:**
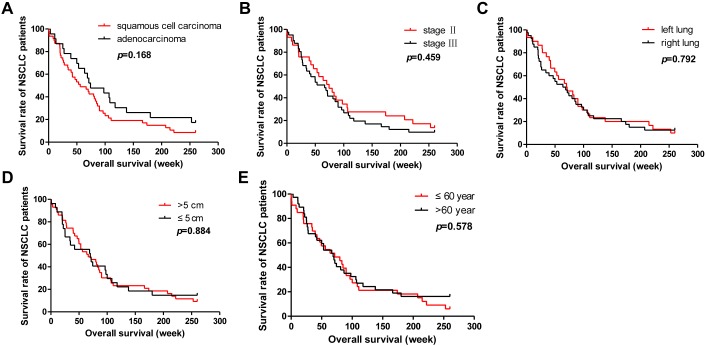
Analysis of the correlation between clinical features and 5-year survival rate of NSCLC patients **(A-E)** Patients were grouped according to the clinical features of the NSCLC patients, pathological type (A), pathological stage (B), pathological location (C), tumor size (D), onset age (E).

Median survival time of the patients in our study was 70.1 weeks and the 5-year survival rate was 11.4%. Univariate and multivariate analysis were performed to study the impact of the target genes expression level and the patients’ clinical features on the survival time of NSCLC patients. Univariate Cox regression analysis showed that the expression level of BRCA1 and STMN1 were significantly correlated with the 5 year survival rate of NSCLC patients (*p*=0.002 and *p*=0.006, Table 2). Kaplan Meier curve analysis was used to study the correlation between the expression of target genes and the survival time of patients (Figure 3). Our study showed that BRAC1 high expression patients had longer survival time (Figure 3A). In contrast, STMN1 lower expressionpatients got longer survival time (Figure 2B). However, the expression level of MAPT and TUBB3, tumor size, tumor location, age, pathological type, clinical stage were not significantly correlated with the survival time of patients (Table 2, Figure 3 and 4). Multivariate Cox regression analysis showed that BRCA1 and STMN1 were independent predictors of prognosis of NSCLC patients(*p*=0.008 and *p*=0.022, Table 2). This study also found that patients with positive BRCA1 expression and negative STMN1 expression had better prognosis (Figure 3E).

**Table 1:** Demographic and clinicopathological parameters of patients (n = 70)

Characteristic	Patients	
	No.	%
Age (years)		
Median	61	
Range	31-79	
Gender		
Male	61	87
Female	9	13
median survival time (week)	70.1	
5-year survival rate	8	11.4
Tumor (cm)		
>5	27	39
≤5	43	61
Pathological location		
Left lung	40	57
Right lung	30	43
Pathological type		
Adenocarcinoma	23	33
Squamous cell carcinoma	47	61
Clinical stage		
II	29	41
III	41	59
Surgery		
Yes	70	100
No	0	0
Postoperative chemotherapy		
Yes	70	100
No	0	0
Smoking history		
Yes	19	27
No	51	73

**Table 2:** Univariate and multivariate analysis of overall survival in 70 NSCLC patients

Variables	Univariate analyses		Multivariate analyses	
	Hazard ratio (95%CI)	*p*-value	Hazard ratio (95%CI)	*p*-value
Age	1.203 (0.451-1.663)	0.469		
Tumor size	1.039 (0.621-1.738)	0.884		
Tumor location	0.935 (0.566-1.543)	0.792		
Pathological type	1.435 (0.859-2.398)	0.168		
Clinical stage	0.827 (0.450-1.368)	0.459		
BRCA1 expression	2.259 (1.343-3.800)	0.002*	2.101 (1.214-53.637)	0.008*
STMN1 expression	0.494 (0.297-0.819)	0.006*	0.528 (0.306-0.913)	0.022*
MAPT expression	0.782 (0.473-1.294)	0.914		
TUBB3 expression	0.877 (0.519-1.482)	0.623		

*p<0.05, statistically significant.

## DISCUSSION

In the past decades, significant progress has been made in the detection and treatment of lung cancer. However, lung cancer remains high morbidity and mortality. NSCLC as the most common type of lung cancer has been widely studied in recent years. Platinum and paclitaxel are commonly used for NSCLC treatment [5–8]. However, the chemotherapy results of NSCLC patients are highly heterogeneous. The drug resistance of tumor is the main factor influencing the effect of chemotherapy [22]. Therefore, it is very necessary to find new biomarkers which can evaluate the sensitivity or toxic side effects of anticancerdrugs. The expression level of BRCA1, STMN1, MAPT and TUBB3 were reported to be closely associated with the clinical effect of patients undergoing chemotherapy [13–16]. This study investigated the correlation between the expression levels of BRCA1, STMN1, MAPT and TUBB3 and the clinical features of the patients. We also study the impact of the target genes expression level and the patients’ clinical features on the survival time of NSCLC patients.

We detectedthat the protein and mRNA expression levels of BRCA1 and MAPT in the cancer tissues were significant lower than that in the adjacent non-tumor tissues (*p*<0.001). In contrast, the protein and mRNA expression levels of STMN1 and TUBB3 in the cancer tissues were significant higher than that in the adjacent non-tumor tissues (*p*≤0.001). Meanwhile, there was no significant correlation (*p*>0.05) between the expression levels of BRCA1, STMN1, MAPT, TUBB3 and the age of the patient or the size and the pathological type of the tumor. The pathological stages of the patients had no significant (*p*>0.05) effect on the expression level of BRCA1, STMN1 and TUBB3. However, the expression of MAPT was significant higher (*p*<0.05) in the patients with stageIII than patients with stage II.

Kaplan–Meier survival analysis showed that STMN1 positive was significantly (*p*=0.006) associated with a poorer prognosis of NSCLC patients who received postoperative adjuvant chemotherapy, which was consistent with our results. Reyes reported that high STMN1 expression was a marker for clinical outcome in endometrial cancer [23]. Bai showed that high STMN1 level was associated with chemo-resistance and poor prognosis in gastric cancer patients [24]. Ueyama announced that high expression of STMN1 is a strong prognosis marker in oral squamous cell carcinoma patients treated by docetaxel-containing regimens [25]. On the contrary, BRCA1 positive was correlated with a better prognosis of NSCLC patients who received postoperative adjuvant chemotherapy (*p*=0.002). Wang's [26] study in NSCLC exhibited that patients with high BRCA1 expression had longer progression free survival (PFS) and overall survival (OS) Quinn's [27] study showed that overall median survival for higher-BRCA1-expressing patients was found to increase following taxane-containing chemotherapy. The results suggest that NSCLC patients with BRCA1 negative or patients with STMN1 positive may have poorer prognosis and will need receiving more comprehensive treatment. However, multivariate Cox analysis indicated that BRCA1 was an independent predictor of 5-year survival rate of NSCLC patients (*p*=0.034), but STMN1 (*p*=0.141) was not.However, we found that the 5-year survival rate of NSCLC patients with BRCA1 positive and STMN1 negative was significantly higher than other patients (*p*<0.001, Figure 3).

In conclusion, our study found that the expression levels ofTUBB3 and MAPT were not associated with the prognosis of NSCLC patients. But the expression levels of BRCA1 and STMN1 were related to the prognosis of NSCLC patients, BRCA1 and STMN1 were independent predictors. It is exciting that BRCA1 combined with STMN1 can also be used as a predictor for NSCLC patients.So, we recommend that the expression levels of BRCA1 and STMN1 should be detected before NSCLC patients received platinum and paclitaxel treatment.

## MATERIALS AND METHODS

### Patients

The study was conducted from June 2009 to July 2011, paraffin-embedded specimen of 70 cases of NSCLC tissues and adjacent non-tumor tissues, in the Hanzhong 3201 hospital, Shaanxi Province. The demographic and clinicopathological parameters of patients were displayed in Table 1. All specimens included in this study were identified by the experienced pathologists. The histological classification and grading were performed according to thestandards of National Comprehensive Cancer Network (NCCN). All patients underwent surgical treatment and postoperative adjuvant chemotherapy (75 mg/m^2^ cisplatin plus 75 mg/m^2^ docetaxel or 500 mg/m^2^ pemetrexed chemotherapy every three weeks for four cycles). This study was approved by the Ethics Committee of Hanzhong 3201 hospital.

### Immunohistochemical analysis

A standard protocol was used for the immunohistochemistry (IHC) of the samples that were detected as NSCLC by hematoxylin and eosin staining. Briefly, formalin fixed, paraffin embedding, paraffin-embedded specimens, dewaxing to water, antigen repair, serum blocking, primary antibody incubation (BRCA1 antibody, Santa Cruz, SC-624; STMN1 antibody, CST, #13655; MAPT antibody, CST,#4019; TUBB3 antibody, CST, #5666), secondary antibody incubation, coloration, counterstaining, dehydration, block.

Each tissue specimen was evaluated independentlyby two pathologists, and eight random fields were used to assess the expression levels of BRCA1, STMN1, MAPT and TUBB3, and also to calculate an average score. In addition, the two pathologists were blinded to the clinical status of the patients. For each patient specimen, these biomarkers were assessed by distribution and intensity. The staining distribution of target proteins were evaluated with the percentage of stained cells, which was scored as 0-100 (low expression: 0-25, high expression: 26-100).

### Western blotting

Fresh tissues were ground broken at low temperature and lysed for 20 min in ice-cold RIPA lysis buffer supplemented with 1 mM PMSF and a cocktail of protease inhibitors. Blotting was performed with antibodies against BRCA1 (Clone # SC-624, Santa Cruz), STMN1 (Cat.#13655, CST), MAPT (Cat.#4019, CST), TUBB3 (Cat.#5666, CST). Goat anti-rabbit and goat anti-mouse immunoglobulin horseradish peroxidase-linked F(ab)2 fragments from Millipore (Billerica, MA, USA) were used as secondary antibodies.

### Quantitative reverse transcriptase PCR (qRT-PCR)

Total RNA was extracted using RNAiso Plus(Takara, #9109, Kusatsu, Shiga, Japan) according to themanufacturer's instructions. Reverse transcription for geneexpression was performed using the PrimeScript™ RTMaster Mix (Takara, #RR036A). qRT-PCR was performedusing the SYBR Green dye (Takara, #RR820A) accordingto the manufacturer's protocol. The following paired primerswere used: BRCA1, F: 5′-AGGTCCAAAGCGAGCAAGAG-3′ and R: 5′-TGCCAAGGGTGAATGATGAA-3′; STMN1, F: 5′-TCTCAGCCCTCGGTCAAA-3′ and R: 5′-GGGACTTGCGTCTTTCTT-3′; TUBB3, R: 5′-GGATTCGGTCCTGGATGTG-3′ and R: 5′ TGATGCGGTCGGGATACTC 3′;MAPT, F: 5′-CCCTGGCGGAGGAAATAA-3′ and R: 5′-TTGCTGAGATGCCGTGGA-3′;β-actin, F: 5′-CGGGAAATCGTGCGTGAC-3′ andR: 5′-CAGGAAGGAAGGCTGGAAG-3′.

### Statistical analysis

All statistical analyses were carried out using StatisticalProgram for Social Sciences (SPSS) software 17.0 (SPSSInc, USA). Paired t test was used to detect the expression of BRCA1, STMN1, MAPT and TUBB3 protein in cancer tissues and adjacent non-tumor tissues. The relationship between the expression level of target protein and the clinicopathological characteristics of the patients were analyzed by chi square test. Survival analysis was performed by Kaplan–Meier method and compared by log-rank test. Factors with significant influence on univariate analysis were further analyzed by multivariate Cox regression analysis. The minimum level of significance was established at *p*<0.05. Statistical analyses were performed using SPSS 19.0 software.
